# Comparative mitogenomics supports synonymy of the genera *Ligula* and *Digramma* (Cestoda: Diphyllobothriidae)

**DOI:** 10.1186/s13071-018-2910-9

**Published:** 2018-05-30

**Authors:** Wen X. Li, Pei P. Fu, Dong Zhang, Kellyanne Boyce, Bing W. Xi, Hong Zou, Ming Li, Shan G. Wu, Gui T. Wang

**Affiliations:** 1 Key Laboratory of Aquaculture Disease Control, Ministry of Agriculture, and State Key Laboratory of Freshwater Ecology and Biotechnology, Institute of Hydrobiology, Chinese Academy of Sciences, Wuhan, 430072 People’s Republic of China; 2 University of Chinese Academy of Sciences, Beijing, 100049 People’s Republic of China; 3 South Devon College University Centre, Long Road, Paignton, TQ4 7EJ UK; 4 Key Laboratory of Freshwater Fisheries and Germplasm Resources Utilization, Ministry of Agriculture, Freshwater Fisheries Research Center, Chinese Academy of Fishery Sciences, Wuxi, 214081 China

**Keywords:** Mitogenome, Eucestoda, Diphyllobothriidea, *Digramma*, *Ligula*

## Abstract

**Background:**

After observing differences in the number of reproductive complexes per proglottid within the genus *Ligula*, the genus *Digramma* was erected. However, the validity of *Digramma* has been previously questioned due to a low variability in the *cox*1, *nad*1 and ITS rDNA sequences between the two genera. We undertook a study to greatly increase the amount of sequence data available for resolution of this question by sequencing and characterizing the complete mitogenomes of *Digramma interrupta* and *Ligula intestinalis*.

**Results:**

The circular mtDNA molecules of *Digramma interrupta* and *Ligula intestinalis* are 13,685 bp and 13,621 bp in size, respectively, both comprising 12 PCGs, 22 tRNA genes, two rRNA genes, and two mNCRs. Both mitogenomes exhibit the same gene order and share 92.7% nucleotide identity, compared with 85.8–86.5% to the most closely related genus *Dibothriocephalus*. Each gene from *D. interrupta* and *L. intestinalis* is almost of the same size, and the sequence identity ranges from 87.5% (*trnD*) to 100% (*trnH*, *trnQ* and *trnV*). NCR2 sequences of *D. interrupta* and *L. intestinalis* are 249 bp and 183 bp in length, respectively, which contributes to the main difference in length between their complete mitogenomes. A sliding window analysis of the 12 PCGs and two rRNAs indicated nucleotide diversity to be higher in *nad*5, *nad*6, *nad*2, *nad*4 and *cox*3, whereas the most conserved genes were *rrnL* and *rrnS*. Lower sequence identity was also found in *nad*2, *nad*4, *nad*5, *nad*6 and *cox*3 genes between the two diphyllobothriids. Within the Diphyllobothriidae, phylogenetic analysis indicated *Ligula* and *Digramma* to be most closely related to one another, forming a sister group with *Dibothriocephalus*.

**Conclusions:**

Owing to higher nucleotide diversity, the genes *nad*2, *nad*4, *nad*5, *nad*6 and *cox*3 should be considered optimal candidates to use as molecular markers for population genetics and species identification between the two closely related species. The phylogenetic results in combination with the comparative analysis of the two mitogenomes, consistently support the congeneric status of *L. intestinalis* and *D. interrupta*.

**Electronic supplementary material:**

The online version of this article (10.1186/s13071-018-2910-9) contains supplementary material, which is available to authorized users.

## Background

Based upon its paraphyly, differences in the position of the genital pore, the presence of an external seminal vesicle and the absence of a uterine sac in the Diphyllobothriidea Kuchta, Scholz, Brabec & Bray, 2008, the order Pseudophyllidea van Beneden in Carus, 1863 was suppressed and the Diphyllobothriidea and Bothriocephalidea Kuchta, Scholz, Brabec & Bray, 2008 were proposed [1–3].

The order Diphyllobothriidea includes 70 species considered valid, classified into 18 genera across three families [2, 4]. Adult diphyllobothriideans are found only in tetrapods, never having been recorded in fish [2], and the plerocercoids of groups such as *Spirometra*, *Diphyllobothrium* Cobbold, 1858 (syn. *Diplogonoporus* Lönnberg, 1892) and *Dibothriocephalus* Lühe, 1899 (a recently resurrected genus including some species from *Diphyllobothrium*, i.e. *Dib. dendriticus* (Nitzsch, 1824), *Dib. nihonkaiensis* (Yamane, Kamo, Bylund & Wikgren, 1986) and *Dib. latus* (Linnaeus, 1758) belonging to the family Diphyllobothriidae are often the principal agents of food-borne cestodosis [5]. *Ligula* spp. also belong to the Diphyllobothriidae; these species use copepods as first intermediate hosts, freshwater fish as second intermediate hosts and birds as definitive hosts [6]. *Ligula intestinalis* (Linnaeus, 1758) is a tapeworm of veterinary importance worldwide that reduces the fecundity of the cyprinid fishes by parasitic castration [6, 7] and induces mass mortalities of the carp *Chanodichthys erythropterus* [8].

Cholodkovsky [9] erected the genus *Digramma* Cholodkovsky, 1915 after observing differences in the number of reproductive complexes contained within each proglottid when studying the genus *Ligula* Linnaeus, 1758. In China, *Ligula* spp. are distributed in the Qinghai-Tibet Plateau with Schizothoracinae fishes serving as the primary second intermediate host. *Digramma* spp. are found across the rest of China where the goldfish *Carassius auratus* acts as their common second intermediate host [10]. However, the validity of *Digramma* has been questioned due to a low level of difference between the species of two genera in the *cox*1, *nad*1 and ITS rDNA sequences [11–13]. Thus *Digramma* is considered to be synonymous with *Ligula* [2]. Owing to the fact that only one gene and a limited number of isolates were included in that study, more sequence data and a greater range of taxa from different genera are required for a more comprehensive phylogenetic analysis of the Diphyllobothriidea [13].

This study, therefore, aimed to sequence and characterize the complete mitogenomes of *Digramma interrupta* and *Ligula intestinalis* and to perform phylogenetic analysis to investigate whether or not these two diphyllobothriids are congeneric using mitogenomic data. Differences within the mitochondrial genes were also compared to determine which genes would be suitable for the design of molecular markers as a means to differentiate *D. interrupta* from *L. intestinalis*.

## Methods

### Specimen collection and DNA extraction

Plerocercoids of *D. interrupta* and *L. intestinalis* were isolated from the body cavity of *Carassius auratus* collected from Liangzi Lake in Hubei Province and *Gymnocypris selincuoensis* from Siling Lake, Tibet, China, respectively. The tapeworms were preserved in 80% ethanol and stored at 4 °C. Total genomic DNA was extracted from the posterior region of a single tapeworm using a TIANamp Micro DNA Kit (Tiangen Biotech, Beijing, China), according to the manufacturer’s instructions. DNA was stored at -20 °C for subsequent molecular analysis. The morphological identification of specimens was confirmed using sequence data from the ITS2 rDNA region [13, 14] and the *cox*1 gene [11].

### Amplification and DNA sequencing

PCR was carried out as described previously [15, 16], with minor modifications. Five degenerate primer sets (Additional file 1: Table S1) were designed to primarily amplify partial sequences of the *nad*5, *cytb*, *nad*2, *cox*1 and *rrnS* genes. The sequenced fragments were subsequently used to design primers specific for the amplification and sequencing of the whole mitogenome. PCR reactions were performed in a 20 μl reaction mixture, containing 7.4 μl dd PCR grade H_2_O, 10 μl 2× PCR buffer (2 mM Mg^2+^, 8 μl dNTP plus, Takara, Dalian, China), 0.6 μl of each primer (12.5 μM), 0.4 μl rTaq polymerase (250 U, Takara, Dalian, China), and 1 μl genomic DNA template. Amplification was conducted under the following conditions: initial denaturation at 98 °C for 2 min, followed by 40 cycles at 98 °C for 10 s, 48–60 °C for 15 s, 68 °C for 1 min/kb, and a final extension at 68 °C for 10 min. PCR products were sequenced on an ABI 3730 automatic sequencer using the Sanger method at Sangon Company (Shanghai, China) using the primer walking strategy.

### Sequence annotation and analyses

The mitogenome was annotated broadly following the procedure described previously [15, 16]. The amplified fragments were initially checked by BLASTN [17], before being assembled manually in a stepwise manner. The annotation was recorded in a Word document with the help of the Geneious program [18], using the mitogenome of *Dibothriocephalus latus* (syn. *Diphyllobothrium latum*) (NC_008945) as the reference sequence. PCGs were found by searching for ORFs (employing genetic code 9, echinoderm mitochondrial; flatworm mitochondrial) and the nucleotide alignments against the selected reference genome in Geneious. *rrnL* and *rrnS* were annotated in a similar way, *via* comparison with homologs using Geneious. ARWEN [19] and MITOS [20] web servers were employed to identify and fold all tRNAs. Similarly, the NCBI submission file (*.sqn) and tables of statistics for mitogenomes were generated using a home-made GUI-based program, MitoTool [21]. MitoTool was also used to calculate codon usage and relative synonymous codon usage (RSCU) for the 12 PCGs of *D. interrupta* and *L. intestinalis* and the results were sorted and imported into ggplot2 [22] to draw the RSCU figure. A Tandem Repeats Finder [23] was used to predict tandem repeats (TR) in the major non-coding regions (mNCRs), and the secondary structures of NCR1 and TR were folded by Mfold software [24]. Non-synonymous (dN) and synonymous (dS) mutation rates among the 12 PCGs of *D. interrupta* and *L. intestinalis* were computed using KaKs_Calculator [25] utilising a modified Yang-Nielsen algorithm. DnaSP v5 [26] was adopted to conduct sliding window analyses. A sliding window of 500 bp and step size of 25 bp was implemented to estimate nucleotide divergence Pi (π) between the alignments of the mitogenomes of *D. interrupta* and *L. intestinalis*.

### Phylogenetic analyses

The mitogenomes of 35 cestodes, covering five orders and ten families (Additional file 2: Table S2) were obtained from GenBank and were used, along with the two new mitogenomes generated in this study, to create the phylogenetic reconstruction. Two trematodes, *Dicrocoelium chinensis* (NC_025279) and *Dicrocoelium dendriticum* (NC_025280), were used as outgroups. All 36 genes (12 PCGs, 2 rRNAs and 22 tRNAs) were used for phylogenetic inference and were extracted from GenBank files using MitoTool. PCGs were aligned in batches using MAFFT and integrated into our own in-house GUI-based program, BioSuite [27], adopting codon-alignment mode. All RNA genes (rRNA and tRNA) were aligned using a structural alignment algorithm Q-INS-i incorporated into MAFFT-with-extensions software [28]. Gaps and ambiguous sites were deleted using GBlocks [29] integrated by BioSuite with default settings. BioSuite was subsequently used to concatenate the sequences into a single alignment and generate phylip and nexus format files.

GTR+I+G was chosen as the optimal model of nucleotide evolution for all datasets based on the Akaike information criterion by ModelGenerator [30]. Two analytical methods were performed: maximum likelihood (ML) and Bayesian inference (BI). The ML analysis was performed in RAxML GUI [31] using a ML+rapid bootstrap (BP) algorithm with 1000 replicates. BI analysis was performed in MrBayes 3.2.6 [32] with default settings, 6 × 10^6^ metropolis-coupled MCMC generations, and 1000 sample frequency. Stationarity was considered to have been reached when the average standard deviation of split frequencies was below 0.01, ESS (estimated sample size) was above 200, and PSRF (potential scale reduction factor) approached 1. Bayesian posterior probability (BPP) values were calculated in a consensus tree, after discarding the first 25% of samples as burn-in. Finally, the resultant trees were visualised and annotated by iTOL [33] with the help of several dataset files generated by MitoTool.

## Results

### Genome organization and base composition

**Fig. 1:**
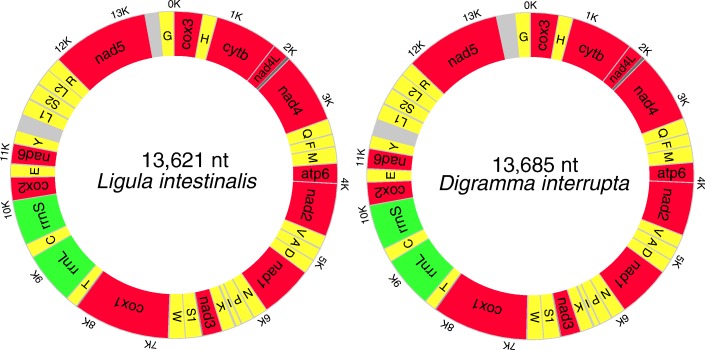
Circular map of the mitochondrial genome of *Digramma interrupta* and *Ligula intestinalis*. Protein-coding genes (12) are shown in red, tRNAs (22) in yellow, rRNAs (2) in green and non-coding regions in grey

The length of the circular mtDNA molecules of *D. interrupta* (GenBank accession number: MF671697) and *L. intestinalis* (GenBank accession number: MF671696) was 13,685 bp and 13,621 bp, respectively. Both mitogenomes were composed of 12 PCGs, 22 tRNA genes, two rRNA genes and two mNCRs (major non-coding regions), all of which were transcribed from the same strand (Fig. 1). As commonly reported for flatworms [34], the two mitogenomes lacked the *atp*8 gene. The gene order of the two mitogenomes was identical, conforming to the synapomorphic gene arrangement of the order Diphyllobothriidea [35]. The A+T content of the mitogenomes of *D. interrupta* and *L. intestinalis* were 67.9% and 67%, respectively, which is in accordance with that of other cestodes (Additional file 2: Table S2).

The mitogenomes of *D. interrupta* and *L. intestinalis* shared 92.7% nucleotide identity (Table 1), compared with 85.8% and 86.2% to *Dib. latus*, 86.1% and 86.5% to *Dib. nihonkaiensis*, species of the most closely related genus, respectively. Nucleotide identity was 92.3% between *Dib. latus* and *Dib. nihonkaiensis*, 99.2% between *Diphyllobothrium grandis* (Blanchard, 1894) and *D. balaenopterae* (Lönnberg, 1892), 99.3% between *Spirometra decipiens* (Diesing, 1850) and *S. erinaceieuropaei* (Rudolphi, 1819), respectively. Amongst all 36 genes, the majority were equal in size between *D. interrupta* and *L. intestinalis*, with the exception of *rrnS*, *trnY* and *trnS2* which had only one base difference. Sequence identity ranged from 87.5% (*trnD*) to 100% (*trnH*, *trnQ* and *trnV*) (Table 1). Seven overlapping regions and 20 intergenic sequences (Gap1–20) were found in both genomes, identical in size and position, with the exception of GAP9, GAP11 and GAP17, which differed in size (Table 1).

**Table 1:** Annotated mitochondrial genomes of *Digramma interrupta* and *Ligula intestinalis*

Gene	Position		Size (bp)	Intergenic nucleotides	Codon		Identity (%)	Sequence
	From	To			Start	Stop		
*Digramma interrupta*/*Ligula intestinalis*								
*cox*3	1/1	643/643	643/643		GTG/GTG	T/T	91.29	
*trnH*	644/644	710/710	67/67				100	
GAP1	711/711	713/713	3/3				100	gaa/gaa
*cytb*	714/714	1820/1820	1107/1107		ATG/ATG	TAA/TAA	93.41	
GAP2	1821/1821	1821/1821	1/1				100	c/c
*nad*4*L*	1822/1822	2082/2082	261/261		ATG/ATG	TAA/TAA	94.64	
*nad*4	2043/2043	3293/3293	1251/1251	-40/-40	ATG/ATG	TAG/TAG	91.37	
*trnQ*	3293/3293	3357/3357	65/65	-1/-1			100	
*trnF*	3353/3353	3419/3419	67/67	-5/-5			97.01	
*trnM*	3416/3416	3482/3482	67/67	-4/-4			98.51	
GAP3	3483/3483	3485/3485	3/3				66.67	gtt/att
*atp*6	3486/3486	3995/3995	510/510		ATG/ATG	TAA/TAA	93.33	
GAP4	3996/3996	3997/3997	2/2				100	tc/tc
*nad*2	3998/3998	4876/4876	879/879		ATG/ATG	TAG/TAG	91.24	
GAP5	4877/4877	4877/4877	1/1				100	t/t
*trnV*	4878/4878	4941/4941	64/64				100	
GAP6	4942/4942	4949/4949	8/8				87.5	gtcttaag/gttttaag
*trnA*	4950/4950	5010/5010	61/61				98.36	
GAP7	5011/5011	5013/5013	3/3				100	tgg/tgg
*trnD*	5014/5014	5077/5077	64/64				87.5	
*nad*1	5078/5078	5968/5968	891/891		ATG/ATG	TAG/TAG	93.04	
*trnN*	5968/5968	6033/6033	66/66	-1/-1			96.97	
GAP8	6034/6034	6040/6040	7/7				100	tatgggt/tatgggt
*trnP*	6041/6041	6105/6105	65/65				95.38	
GAP9	6106/6106	6112/6113	7/8				62.5	cgcatta/tagtatta
*trnI*	6113/6114	6177/6178	65/65				93.85	
GAP10	6,178/6,179	6194/6195	17/17				82.35	taaagaaggaaaggata/taaagaaggaaaaggtg
*trnK*	6195/6196	6258/6259	64/64				98.44	
GAP11	6259/6260	6261/6260	3/1				33.33	aat/a
*nad*3	6262/6261	6618/6617	357/357		ATG/ATG	TAG/TAG	94.96	
*trnS1*	6608/6607	6666/6665	59/59	-11/-11			96.61	
GAP12	6667/6666	6668/6667	2/2				50	tc/tt
*trnW*	6669/6668	6731/6730	63/63				93.65	
GAP13	6732/6731	6739/6738	8/8				87.5	aatataaa/agtataaa
*cox*1	6740/6739	8305/8304	1566/1566		ATG/ATG	TAG/TAG	93.49	
*trnT*	8296/8295	8357/8356	62/62	-10/-10			98.39	
*rrnL*	8358/8357	9324/9323	967/967				96.38	
*trnC*	9325/9324	9388/9387	64/64				98.44	
*rrnS*	9389/9388	10130/10130	742/743				95.83	
*cox*2	10131/10131	10700/10700	570/570		ATG/ATG	TAA/TAA	94.39	
GAP14	10701/10701	10701/10701	1/1				100	a/a
*trnE*	10702/10702	10765/10765	64/64				98.44	
GAP15	10766/10766	10770/10770	5/5				100	ttagc/ttagc
*nad*6	10771/10771	11229/11229	459/459		ATG/ATG	TAG/TAG	91.29	
GAP16	11230/11230	11232/11232	3/3				100	ata/ata
*trnY*	11233/11233	11298/11297	66/65				96.97	
*NCR1*	11299/11298	11521/11521	223/224				91.07	
*trnL1*	11522/11522	11588/11588	67/67				91.04	
GAP17	11589/11589	11600/11601	12/13				84.62	tgcggggggttt/ttgtggggggttt
*trnS2*	11601/11602	11665/11667	65/66				96.97	
GAP18	11666/11668	11676/11678	11/11				72.73	tagttaaaaga/cagttaaataa
*trnL2*	11677/11679	11740/11742	64/64				95.31	
*trnR*	11741/11743	11795/11797	55/55				92.73	
GAP19	11796/11798	11798/11800	3/3				100	ttt/ttt
*nad*5	11799/11801	13367/13369	1569/1569		ATG/ATG	TAA/TAA	90.12	
*NCR2*	13368/13370	13616/13552	249/183				67.86	
*trnG*	13617/13553	13682/13618	66/66				96.97	
GAP20	13683/13619	13685/13621	3/3				100	aag/aag
genome	1/1	13685/13621	13,685/13,621				92.72	

### Protein-coding genes and codon usage

Concatenated PCGs of the mitogenome of *D. interrupta* and *L. intestinalis* were both 10,062 bp in size, with an A+T content of 67.5% and 66.6%, respectively (Table 2). The high A+T content was mainly concentrated on the third codon position (72.4% for *D. interrupta* and 70.1% for *L. intestinalis*). The start and termination codons within the mitogenome of *D. interrupta* and *L. intestinalis* were identical to one another. GTG was identified as the initial codon for *cox*3, and ATG for the rest of the 12 PCGs (Table 1). For each PCG, however, all selected Diphyllobothriidea species shared the same start codon (Additional file 3: Table S3), indicating that it may be a synapomorphy within this order. Amongst the termination codons, five out of 12 were identified as TAA, six as TAG, while *cox*3 used a truncated T stop codon.

**Table 2:** Nucleotide composition and skewness of different elements of the mitochondrial genomes of *Digramma interrupta* and *Ligula intestinalis*

Region	Size (bp)	T(U)	C	A	G	AT (%)	GC (%)	AT skew	GC skew
*Digramma interrupta*/*Ligula intestinalis*									
PCGs	10,062/10,062	45.6/45.1	12.3/13.1	21.9/21.5	20.2/20.3	67.5/66.6	32.5/33.4	-0.351/-0.354	0.244/0.216
1st codon position	3354/3354	41.2/41.4	11.2/11.3	23.6/23.4	24.0/23.9	64.8/64.8	35.2/35.2	-0.272/-0.277	0.366/0.358
2nd codon position	3354/3354	47.6/47.3	15.1/15.4	17.6/17.5	19.7/19.7	65.2/64.8	34.8/35.1	-0.461/-0.461	0.132/0.122
3rd codon position	3354/3354	47.9/46.5	10.6/12.6	24.5/23.6	17.0/17.4	72.4/70.1	27.6/30.0	-0.323/-0.327	0.231/0.159
*atp*6	510/510	47.6/47.3	13.3/13.3	21.6/20.2	17.5/19.2	69.2/67.5	30.8/32.5	-0.377/-0.401	0.134/0.181
*cox*1	1566/1566	45.0/44.0	12.6/13.8	22.3/21.8	20.1/20.4	67.3/65.8	32.7/34.2	-0.336/-0.338	0.229/0.194
*cox*2	570/570	40.2/38.9	12.8/14.2	23.9/24.7	23.2/22.1	64.1/63.6	36.0/36.3	-0.255/-0.223	0.288/0.217
*cox*3	643/643	46.8/47.1	12.4/12.4	20.5/19.4	20.2/21.0	67.3/66.5	32.6/33.4	-0.390/-0.416	0.238/0.256
*cytb*	1107/1107	43.8/43.9	13.7/14.1	22.1/21.6	20.3/20.4	65.9/65.5	34.0/34.5	-0.329/-0.341	0.194/0.183
*nad*1	891/891	45.3/44.9	11.0/11.4	20.4/21.1	23.2/22.6	65.7/66.0	34.2/34.0	-0.379/-0.361	0.357/0.327
*nad*2	879/879	48.9/48.9	10.5/11.1	21.0/19.8	19.6/20.1	69.9/68.7	30.1/31.2	-0.398/-0.424	0.303/0.287
*nad*3	357/357	51.3/49.9	7.8/9.2	21.8/20.7	19.0/20.2	73.1/70.6	26.8/29.4	-0.402/-0.413	0.417/0.371
*nad*4	1251/1251	47.2/46.9	13.7/14.2	19.4/19.0	19.6/19.8	66.6/65.9	33.3/34.0	-0.417/-0.423	0.175/0.164
*nad*4*L*	261/261	48.3/49.4	9.2/8.8	27.2/27.2	15.3/14.6	75.5/76.6	24.5/23.4	-0.279/-0.290	0.250/0.246
*nad*5	1569/1569	42.4/41.4	13.3/15.2	23.8/23.6	20.5/19.8	66.2/65.0	33.8/35.0	-0.281/-0.273	0.214/0.133
*nad*6	459/459	48.8/48.1	9.8/10.2	21.1/21.1	20.3/20.5	69.9/69.2	30.1/30.7	-0.396/-0.390	0.348/0.333
*rrnL*	967/967	40.1/39.7	12.0/12.2	28.3/28.2	19.5/19.9	68.4/67.9	31.5/32.1	-0.172/-0.169	0.239/0.239
*rrnS*	742/743	38.0/38.2	12.1/12.9	30.2/29.3	19.7/19.5	68.2/67.5	31.8/32.4	-0.115/-0.131	0.237/0.203
*NCR1*	223/224	44.4/41.5	8.5/10.3	34.5/33.5	12.6/14.7	78.9/75.0	21.1/25.0	-0.125/-0.107	0.191/0.179
*NCR2*	249/183	51.8/50.3	7.6/6.6	22.1/24.6	18.5/18.6	73.9/74.9	26.1/25.2	-0.402/-0.343	0.415/0.478
tRNAs	1410/1410	38.5/37.9	12.4/13.0	29.1/28.6	20.0/20.4	67.6/66.5	32.4/33.4	-0.140/-0.141	0.234/0.220
Full genome	13,685/13,621	44.1/43.6	12.1/12.9	23.8/23.4	20.0/20.2	67.9/67.0	32.1/33.1	-0.299/-0.301	0.245/0.221

Codon usage, RSCU, and codon family proportion (corresponding to the amino acid usage) among *D. interrupta* and *L. intestinalis* were investigated (Additional file 4: Figure S1). The most abundant codon families were Phe, Leu2, and Ile within the two mitogenomes, which show a preference for the A+T-rich synonymous codons (Additional file 4: Figure S1). This corresponds to the high A+T bias of the two diphyllobothriid mitogenomes.

**Fig. 2:**
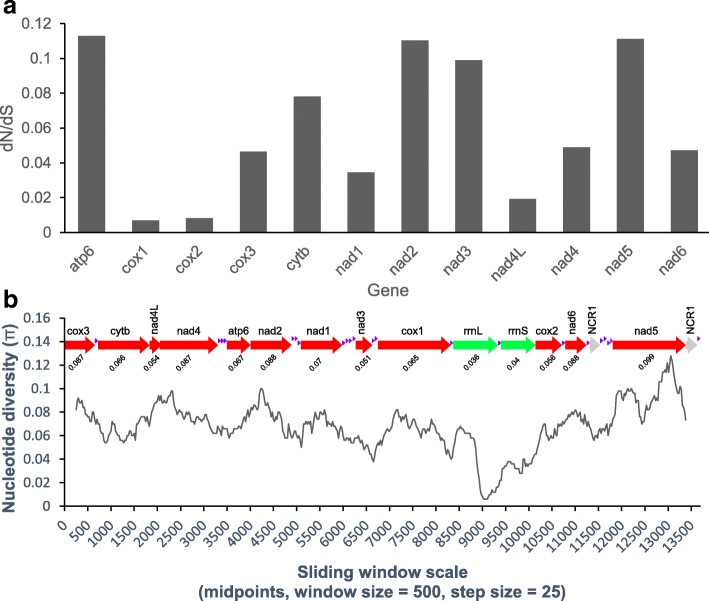
**a** Ratios of non-synonymous (dN) to synonymous (dS) substitution rates estimated from individual protein-coding genes of *Digramma interrupta* and *Ligula intestinalis*. **b** Sliding window analysis of the alignment of complete mtDNAs of *D. interrupta* and *L. intestinalis*. The black line shows the value of nucleotide diversity in a sliding window analysis of window size 500 bp with step size 25 bp, and the value is inserted at its mid-point. Gene boundaries are indicated with a variation ratio per gene (below each gene)

We measured the selective pressure acted upon amino acid replacement mutations by the ratio of non-synonymous (dN) to synonymous (dS) substitutions for all 12 PCGs of *D. interrupta vs L. intestinalis*. Although the values (dN/dS) of *atp*6 (0.113), *nad*5 (0.111) and *nad*2 (0.110) genes were higher than *cox*1 (0.007) and *cox*2 (0.008) genes (Fig. 2a), all PCGs were under strong negative (purifying) selection (dN/dS < 0.12).

### Transfer and ribosomal RNA genes

The sizes of *rrnL* in both the *D. interrupta* and *L. intestinalis* mitogenome was 967 bp, with 68.4% and 67.9% A+T content, respectively. Similarly, the lengths of *rrnS* were 742 and 743 bp, with an A+T content of 68.2% and 67.5%, respectively (Table 2). All 22 commonly found tRNAs were present in the mitochondrial genome of *D. interrupta* and *L. intestinalis*, adding up to a 1,410 bp total concatenated length in both mitogenomes (Table 2). All tRNAs could be folded into the conventional cloverleaf structure, with the exception of *trnS*1^(AGN)^ and *trnR*, which lacked DHU arms. The absence of DHU-arms in *trnS*1^(AGN)^ and *trnR* has also been reported in the Caryophyllidea [35] and the Anoplocephalidae [36].

### Non-coding regions

**Fig. 3:**
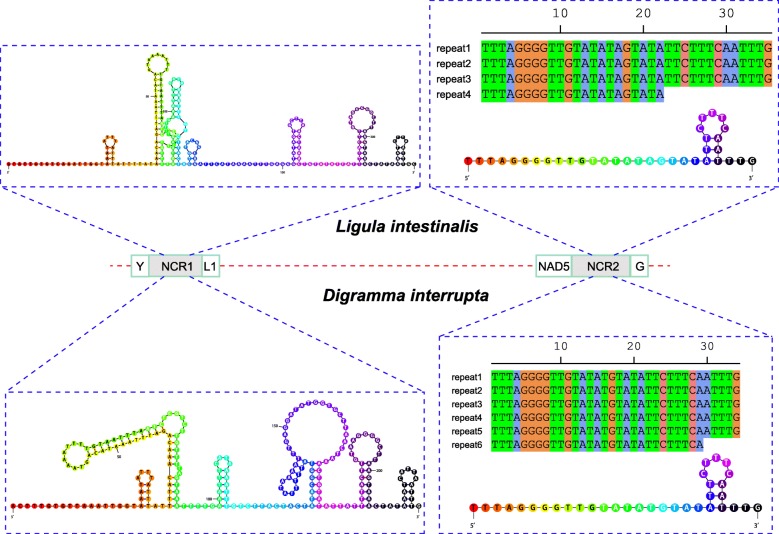
Major non-coding regions (mNCRs) in the mitogenomes of *Digramma interrupta* and *Ligula intestinalis*. Tandem repeat units are shown on the right. The secondary structures of the mNCRs and consensus repeat sequence are illustrated

The two major non-coding regions (mNCRs), NCR1 and NCR2, were located between *trnY* and *trnL*1 and between *nad*5 and *trnG*, respectively. The mNCRs were situated in the same location as all diphyllobothriideans surveyed to date (see Additional file 3 in our recent paper [35]). The NCR1 sequences of the two mitogenomes of *D. interrupta* and *L. intestinalis* were 223 and 224 bp in length with a heightened A+T bias of 78.9% and 75%, whereas the NCR2 sequences were 249 and 183 bp in size with 73.9% and 74.9% A+T content, respectively (Table 2). The NCR2 of *D. interrupta* contained six TRs (tandem repeats). Repeat units 1–5 were identical in nucleotide composition and size (34 bp). Repeat unit 6 was truncated with 29 bp (Fig. 3). TRs were also found in the NCR2 of *L. intestinalis*, and the consensus repeat (35 bp) was almost identical to that of *D. interrupta*, with an insertion of a single nucleotide A at the 17th position. Only four repeat units, however, could be found in the mitogenome of *L. intestinalis*, which contributed to the main difference in length of the complete mitogenome between the two genera. The last repeat unit was also truncated with 22 bp. Both NCR1 and the consensus repeat sequence in NCR2 of the two mitogenomes were capable of forming stem-loop structures (Fig. 3, predicted by Mfold web server).

### Sliding window analyses and nucleotide diversity

A sliding window analysis of the 12 PCGs and two rRNAs of *D. interrupta* and *L. intestinalis* indicated the nucleotide diversity Pi (π) to be higher in *nad*5 (0.099), *nad*6 (0.088), *nad*2 (0.088), *nad*4 (0.087) and *cox*3 (0.087), whereas the most conserved genes were *rrnL* (0.036) and *rrnS* (0.04) (Fig. 2b).

### Phylogeny

**Fig. 4:**
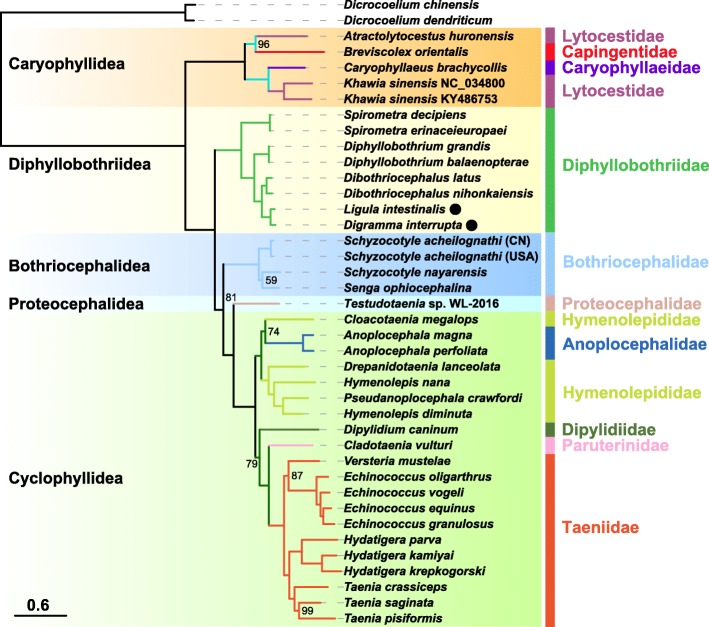
Phylogeny of five cestode orders using maximum likelihood analysis inferred from concatenated nucleotide sequences of all 36 genes (12 PCGs, 2 rRNAs and 22 tRNAs). Bootstrap support values are shown above the nodes. The anti-codon of the *trnR* gene present in individual orders was denoted. The *scale-bar* represents the estimated number of substitutions per site

Both methods (BI and ML) produced phylograms with concordant branch topologies, thus only the latter was shown (Fig. 4). The phylogenetic tree indicated the ordinal topology to be Caryophyllidea + (Diphyllobothriidea + (Bothriocephalidea + (Proteocephalidea + Cyclophyllidea))). Within the family Diphyllobothriidae, *Ligula* and *Digramma* clustered with maximum nodal support (BP = 100 and BPP = 1), which formed a sister group with the genus *Dibothriocephalus*. This clade clustered together with *Diphyllobothrium*, then forming a sister group with *Spirometra*.

## Discussion

The ordinal topology of Caryophyllidea + (Diphyllobothriidea + (Bothriocephalidea + (Proteocephalidea + Cyclophyllidea))) was consistent with previously identified interordinal relationships of tapeworms, when reconstructed from the dataset of nucleotide or amino acid sequences from partial mitogenomes, large and small subunit rRNA genes, and a combination of the former two [37]. Additionally, this relationship identified between tapeworm groups was congruent with latest mitochondrial phylogenomics [35]. However, a sister-group relationship between the orders Diphyllobothriidea and Bothriocephalidea has been suggested based on mitochondrial phylogenomics [38, 39]. This inconsistency may be due to the different methods of phylogenetics employed.

Within the family Diphyllobothriidae, the phylogenetic relationship of the three genera *Spirometra*, *Diphyllobothrium* and *Dibothriocephalus* was congruent with that of recent studies on the phylogenetics of Eucestoda based on mitogenomes, with the topology of *Spirometra* + (*Dibothriocephalus* + *Diphyllobothrium*) [35, 38–41]. In the present study, however, *Ligula* and *Digramma* were closely related to one another with maximum nodal support, then forming a sister group with the genus *Dibothriocephalus*. Inferred by the ITS2 rDNA sequence [13] and the 18S rDNA gene [42], the complexes of *Ligula* and *Digramma* have also been shown to be closely related to *Dibothriocephalus*. Further studies using the concatenated nucleotide sequences of *18S* rDNA + *28S* rDNA + *rrnL* + *cox*1, have again demonstrated the genus *Dibothriocephalus* to be the sister group of *Ligula* [5]. These phylogenetic results suggest that *Dibothriocephalus* is the most closely related genus to *Ligula* and *Digramma*.

However, mitogenome sequence identity between *D. interrupta* and *L. intestinalis* is 92.7%, which is much higher than between either of these species and the represented members of *Dibothriocephalus* (85.8–86.5%). Furthermore, high mitogenome sequence identity was also found between the congeners in *Dibothriocephalus* (92.3%), *Diphyllobothrium* (99.2%) and *Spirometra* (99.3%). These results suggest that sequence differences between *D. interrupta* and *L. intestinalis* are of a degree expected between members of the same genus.

**Fig. 5:**
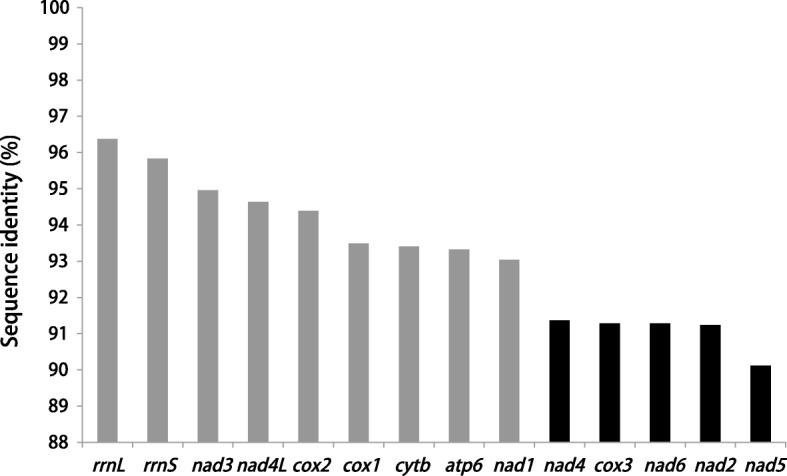
Sequence identity of 12 protein-coding genes and two rRNA genes between *Digramma interrupta* and *Ligula intestinalis*

In one study, sequence identity of the *cox*1 and *nad*1 genes between *D. interrupta* and *L. intestinalis* has been shown to be 100% and 92.6% [11]; however, identity was deemed at 93.5% and 93.0% in the present study, respectively (Table 1). This inconsistency may be due to the use of partial sequence of *cox*1 and *nad*1 genes or resulting from the use of formalin-preserved specimens [12]. The gene *cox*1 is considered to be a useful barcode for metazoans [43], and widely employed for cestode studies [44–47]. The two mitochondrial genes *cox*1 and *cytb* have also been used to study the population genetic structure of *L. intestinalis* on a local and global scale [14]. However, a lower sequence identity was found in the *nad*2, *nad*4, *nad*5, *nad*6 and *cox*3 genes between *D. interrupta* and *L. intestinalis* (90–92%), in comparison to the moderate variation seen between the *cox*1, *cytb* and *nad*1 genes (Fig. 5). Additionally, the relatively looser selection pressure of *nad*5 (0.111) and *nad*2 (0.110) may accelerate the accumulation of non-synonymous substitutions, which would increase variation of the two genes [48]. These results suggest that the *nad*2, *nad*4, *nad*5, *nad*6 and *cox*3 genes should be considered as optimal candidates for genetic markers to be used for population genetics and species identification studies between the two closely related species, *D. interrupta* and *L. intestinalis*.

## Conclusions

The complete mitogenomes of *Ligula intestinalis* and *Digramma interrupta* were sequenced and characterized. The mitogenomes of these two species show a higher identity to each other than to any species in closely related genera. The two mitogenomes consistently support *D. interrupta* to be a congeneric species with *L. intestinalis*. High sequence variation in the *nad*2, *nad*4, *nad*5, *nad*6 and *cox*3 genes between the two diphyllobothriids suggest that these five genes should be considered as optimal candidates for genetic markers when studying population genetics or looking to differentiate the two closely related species, *D. interrupta* and *L. intestinalis*.

## Additional files

Additional file 1: **Table S1.** Primers used to amplify and sequence the mitochondrial genome of *Digramma interrupta* and *Ligula intestinalis*. (DOCX 15 kb)

Additional file 2: **Table S2.** The list of cestode species and outgroups used for comparative mitogenomic and phylogenetic analyses, and accession number, A+T content and skewness of different elements of each mitogenome. (XLSX 19 kb)

Additional file 3: **Table S3.** General statistics (length and codons) for mitochondrial protein-coding genes and rRNAs of 38 cestodes. Abbreviations of species name are the initials of genus and species name combined. (XLSX 21 kb)

Additional file 4: **Figure S1.** Relative Synonymous Codon Usage (RSCU) of *Digramma interrupta* and *Ligula intestinalis*. Codon families are labelled on the x-axis. Values on the top of the bars denote amino acid usage. (PDF 37 kb)

## Abbreviations

dN: Non-synonymous substitutions
dS: Synonymous substitutions
mNCRs: major Non-coding regions
PCGs: Protein-coding genes
