# Supplementary material for: Comparative mitogenomics supports synonymy of the genera Ligula and Digramma (Cestoda: Diphyllobothriidae)
Source: Parasit Vectors. 2018 May 30;11:324. doi: 10.1186/s13071-018-2910-9 (PMC5975392; doi:10.1186/s13071-018-2910-9)
Supplement: Supplementary file 1 — Table S1. Primers used to amplify and sequence the mitochondrial genome of Digramma interrupta and Ligula intestinalis. (DOCX 15 kb) [file 13071_2018_2910_MOESM1_ESM.docx]

Table S1. Primers used to amplify and sequence the mitochondrial genome of *Digramma interrupta* and *Ligula intestinalis*.

| Fragment  No. | Gene or  region | Primer  name | Sequence (5’-3’) | Length  (bp) |
| --- | --- | --- | --- | --- |
| *Digramma interrupta* | | | | |
| F1 | *NAD5* | TCND5F | GAAGCTATGCGDGCACCTACNCC | 470 |
|  |  | TCND5R | TGCTTAGTAAARAANACCCC |  |
| F2 | *NAD5-CYTB* | 1-JF1 | GAAATTGTGTTTACTCTAGATC | 2228 |
|  |  | 1-JR1 | CAAAACCAAGAATAACATGAGC |  |
| F3 | *CYTB* | TCCYTBF | GTCATAYTGRGCTGCTACTGT | 411 |
|  |  | TCCYTBR | AARTACCATTCTGGCTTWAT |  |
| F4 | *CYTB-NAD2* | 1-JF2 | CATTCTTATTTTACTACTAAGG | 2730 |
|  |  | 1-JR2 | CAACCAATGCACAACCTACAC |  |
| F5 | *NAD2* | TCND2F | GTGTTYGTAGCGNTAGTGCAC | 1272 |
|  |  | TCND2R | ACCTTTTTAGGACCCTTACG |  |
| F6 | *NAD2-COX1* | 1-JF3 | GTCTAACCAAAGGCGAAATG | 2503 |
|  |  | 1-JR3 | CCCAAAAGTATCATAAGAAC |  |
| F7 | *COX1* | TCCOX1F | GATCCDTTAGGWGGTGGTGATCC | 666 |
|  |  | TCCOX1R | CACACACGACGTGGTAAACC |  |
| F8 | *COX1-12S* | 1-JF4 | GTTACAGTGTCATTGTATAG | 1727 |
|  |  | 1-JR4 | CCCTTTCATAACTAAACCAC |  |
| F9 | *12S* | TC12SF | CAGTGCCAGCATCCKCGGTTA | 492 |
|  |  | TC12SR | GNTGACGGGCGGTRTGTAC |  |
| F10 | *12S-NAD5* | 1-JF5 | GGTTAACTTTGTAATAAGG | 2606 |
|  |  | 1-JR5 | CTATCTTCTTGAGGTCCAAG |  |
| *Ligula intestinalis* | | | | |
| F1 | *NAD5* | TCND5F | GAAGCTATGCGDGCACCTACNCC | 827 |
|  |  | TCND5R2 | ACHAGGTTRTCTGAGCCAAA |  |
| F2 | *NAD5-COX3* | S4-F1 | CATACCTTGGCTACAGTTG | 944 |
|  |  | S4-R1 | CAGTAATCAAGAATAAGGTC |  |
| F3 | *COX3* | TCCOX3F | GGCTNTTAGTTAGTTGTTTT | 335 |
|  |  | TCCOX3R | ATCCACAAARTGTCAATATC |  |
| F4 | *COX3-CYTB* | S4-F2 | CGTGATTGGCTTAAGTATGG | 735 |
|  |  | S4-R2 | GCCAAGAATAACATGGGCTG |  |
| F5 | *CYTB* | TCCYTBF | GTCATAYTGRGCTGCTACTGT | 411 |
|  |  | TCCYTBR | AARTACCATTCTGGCTTWAT |  |
| F6 | *CYTB-NAD1* | S4-F3 | GTTCAATGTGAGTTGCTCC | 3922 |
|  |  | S4-R3 | CACAACAATTAGAACCAATAAG |  |
| F7 | *NAD1* | TCNAD1F1 | CGTAAGGGNCCAAAHAAGGTTG | 704 |
|  |  | TCNAD1R1 | CGAACHCGTGGCARTGTAGCACG |  |
| F8 | *NAD1-12S* | S4-F4 | TGCTTGTGAGTATATCATC | 3947 |
|  |  | S4-R4 | CCCTTTCATAACTAAACCAC |  |
| F9 | *12S* | TC12SF | CAGTGCCAGCATCCKCGGTTA | 492 |
|  |  | TC12SR | GNTGACGGGCGGTRTGTAC |  |
| F10 | *12S-NAD5* | S4-F5 | CTATGTGCTGCATGCCAAGG | 2621 |
|  |  | S4-R5 | GCAGCAAACTCCCGTAATG |  |
