# Supplementary material for: Comparative mitogenomics supports synonymy of the genera Ligula and Digramma (Cestoda: Diphyllobothriidae)
Source: Parasit Vectors. 2018 May 30;11:324. doi: 10.1186/s13071-018-2910-9 (PMC5975392; doi:10.1186/s13071-018-2910-9)
Supplement: Supplementary file 4 — Figure S1. Relative Synonymous Codon Usage (RSCU) of Digramma interrupta and Ligula intestinalis. Codon families are labelled on the x-axis. Values on the top of the bars denote amino acid usage. (PDF 37 kb) [file 13071_2018_2910_MOESM4_ESM.pdf]

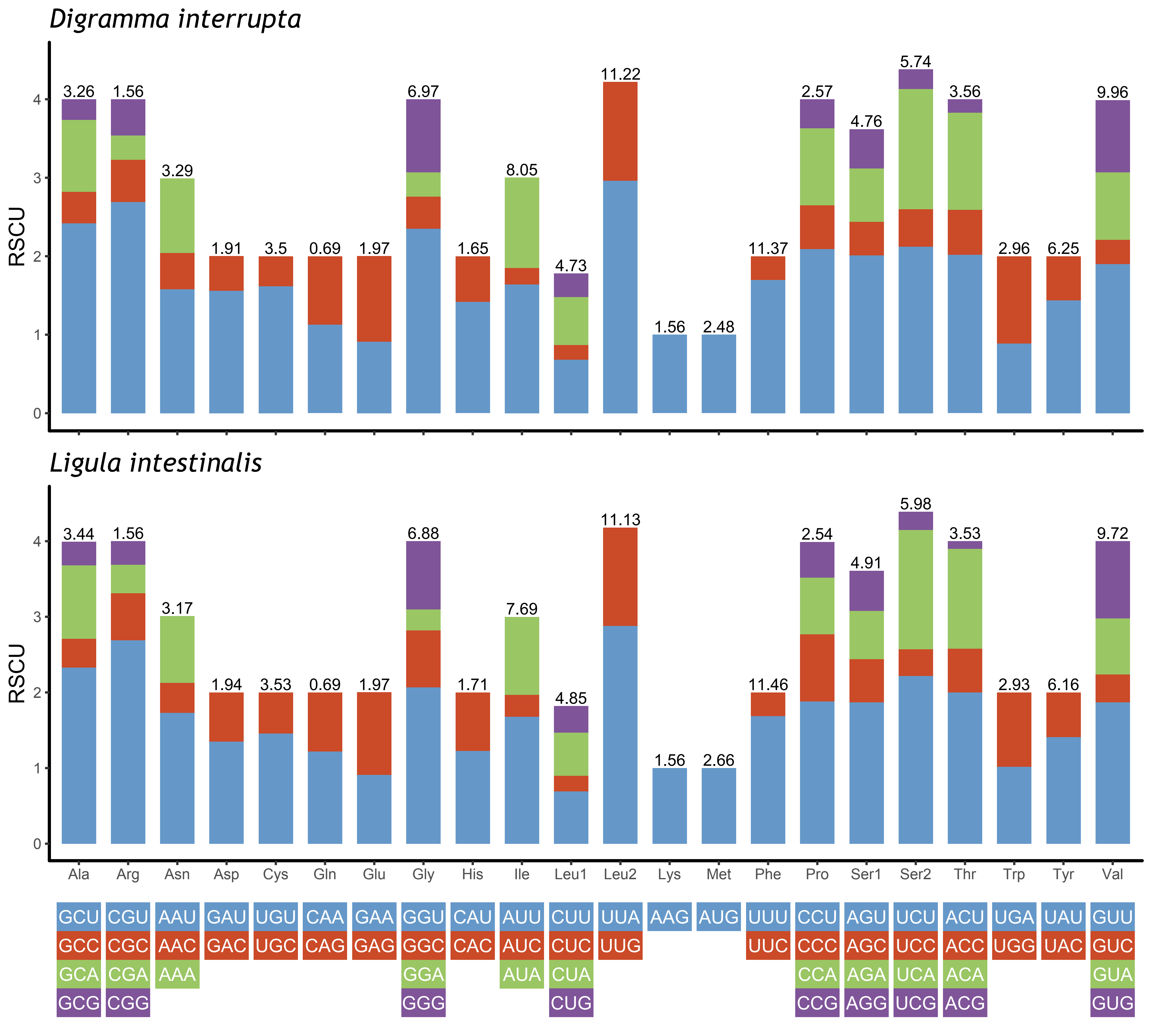
Digramma interrupta
RSCU
0
1
2
3
4
3.26
1.56
3.29
1.91
3.5
0.69
1.97
6.97
1.65
8.05
4.73
11.22
1.56
2.48
11.37
2.57
4.76
5.74
3.56
2.96
6.25
9.96
Ligula intestinalis
RSCU
3.44
1.56
3.17
1.94
3.53
0.69
1.97
6.88
1.71
7.69
4.85
11.13
1.56
2.66
11.46
2.54
4.91
5.98
3.53
2.93
6.16
9.72
Ala
Arg
Asn
Asp
Cys
Gln
Glu
Gly
His
Ile
Leu1
Leu2
Lys
Met
Phe
Pro
Ser1
Ser2
Thr
Trp
Tyr
Val
GCU CGU AAU GAU UGU CAA GAA GGU CAU AUU CUU UUA AAG AUG UUU CCU AGU UCU ACU UGA UAU GUU
GCC CGC AAC GAC UGC CAG GAG GGC CAC AUC CUC UUG UUC CCC AGC UCC ACC UGG UAC GUC
GCA CGA AAA GGA AUA CUA CCA AGA UCA ACA GUA
GCG CGG GGG CUG CCG AGG UCG ACG GUG
